# Morphological and microsatellite DNA diversity of Nigerian indigenous sheep

**DOI:** 10.1186/2049-1891-3-38

**Published:** 2012-11-24

**Authors:** Brilliant O Agaviezor, Sunday O Peters, Mufliat A Adefenwa, Abdulmojeed Yakubu, Olufunmilayo A Adebambo, Michael O Ozoje, Christian ON Ikeobi, Matthew Wheto, Oyeyemi O Ajayi, Samuel A Amusan, Oludotun J Ekundayo, Timothy M Sanni, Moses Okpeku, Gbolabo O Onasanya, Marcos De Donato, Babatunde M Ilori, Kadir Kizilkaya, Ikhide G Imumorin

**Affiliations:** 1 Department of Animal Breeding and Genetics, University of Agriculture, Abeokuta, Nigeria; 2 Department of Animal Science and Fisheries, University of Port Harcourt, Port Harcourt, Nigeria; 3 Dept of Animal Science, Cornell University, Ithaca, NY 14853, USA; 4 Dept of Animal Science, Berry College, Mount Berry, GA 30149, USA; 5 Department of Cell Biology and Genetics, University of Lagos, Lagos, Nigeria; 6 Department of Animal Science, Nasarawa State University, Keffi, Shabu-Lafia Campus, Lafia, Nigeria; 7 Dept of Livestock Production, Niger Delta University, Amasomma, Bayelsa State, Nigeria; 8 Department of Animal Science, Adnan Menderes University, Aydin 09100, Turkey

**Keywords:** Discriminant analysis, Genetic distance, Microsatellite DNA, Morphological traits, Nigerian sheep

## Abstract

**Background:**

Sheep is important in the socio-economic lives of people around the world. It is estimated that more than half of our once common livestock breeds are now endangered. Since genetic characterization of Nigerian sheep is still lacking, we analyzed ten morphological traits on 402 animals and 15 microsatellite DNA markers in 384 animals of the 4 Nigerian sheep breeds to better understand genetic diversity for breeding management and germplasm conservation.

**Results:**

Morphological traits of Uda and Balami were significantly (P < 0.05) higher than Yankasa, which were both higher than West African Dwarf (WAD) sheep. Stepwise discriminant analysis showed tail length, rump height, chest girth, ear length and chest depth as the most discriminating variables for classification. Mahalanobis distances show the least differentiation between Uda and Balami and the largest between WAD and Balami sheep. While 93.3% of WAD sheep were correctly assigned to their source genetic group, 63.9% of Yankasa, 61.2% of Balami and 45.2% of Uda were classified correctly by nearest neighbour discriminant analysis. The overall high Polymorphism Information Content (PIC) of all microsatellite markers ranged from 0.751 to 0.927 supporting their use in genetic characterization. Expected heterozygosity was high for all loci (0.783 to 0.93). Mean heterozygote deficiency across all populations (0.171 to 0.534) possibly indicate significant inbreeding (P < 0.05). Mean values for *F*_*ST*_, F_*IT*_ and F_*IS*_ statistics across all loci were 0.088, 0.394 and 0.336 respectively. Yankasa and Balami are the most closely related breeds (D_A_ = 0.184) while WAD and Balami are the farthest apart breeds (D_A_ = 0.665), which is coincident with distance based on morphological analysis and population structure assessed by STRUCTURE.

**Conclusions:**

These results suggest that within-breed genetic variation in Nigerian sheep is higher than between-breeds and may be a valuable tool for genetic improvement and conservation. The higher genetic variability in Yankasa suggests the presence of unique ancestral alleles reflecting the presence of certain functional genes which may result in better adaptability in more agro-ecological zones of Nigeria. These genetic characteristics are potentially useful in planning improvement and conservation strategies in Nigerian indigenous sheep.

## Background

The population of sheep in Nigeria is currently estimated at 33.9 million making up 3.1% of the world’s total [1]. Sheep is an important livestock species in the socio-economic lives of people around the world including Nigerians [2]. Increased loss of genetic diversity has been observed for all agriculturally used species, and it is estimated that more than half of our once common livestock breeds are now endangered [1]. According to FAO [3], it is estimated that the world loses two breeds of its valuable domestic animal diversity every week. Already, 740 breeds are recorded as extinct, with 1,335 (32% of the estimated total) being classified at high risk of loss or under threat of extinction. If the erosion of animal genetic diversity continues without adequate action, more than 2,000 domestic animal breeds could be lost within the next two decades [3]. The first step toward an efficient conservation strategy for cattle, sheep, and goat genetic resources is the proper characterization of the conservation value of the different breeds and their wild relatives [4].

Sheep biodiversity have been described using morphological measurements [5,6] or characterized using molecular data [7-13]. The phenotypic variation in a population arises due to genotypic and environmental effects, and the magnitude of phenotypic variability differs under different environmental conditions. Morphometric characters are continuous characters describing aspects of body shape [14,15]. Morphometric variation between populations can provide a basis for understanding flock structure, and may be more applicable for studying short-term, environmentally induced variation and thus more applicable to livestock management. According to Gizaw et al. [5], morphological description is an essential component of breed characterization that can be used to physically identify, describe, and recognize a breed, and also to classify livestock breeds into broad categories. Dossa et al. [16] reported that morphological measurements such as heart girth, height at withers and body length can be used for rapid selection of large size individuals in the field to enable the establishment of elite flocks. In addition, microsatellites have been used successfully over the years to characterize the genetic diversity of sheep populations in China [12], Ethiopia [5], Europe and Middle East [7-9,17], India [10,11,18] and Brazil [13].

The Nigerian sheep is still genetically unimproved, and the pressure of modern genetic improvement has increased the need to better understand natural genetic variation in Nigerian sheep breeds, as well as formulate germplasm conservation policies. The only genetic diversity study of genetic variation among Nigerian sheep breeds using microsatellite markers covered a limited geographical area [19]. Therefore, a more detailed study using a larger sample size from across the entire country is still required to better understand the genetic structure of Nigerian sheep population. In this study, morphological data on 402 sheep and molecular data on 15 microsatellite DNA markers in 384 sheep sampled across the entire country were used to evaluate the morphological and genetic diversity of the four major extant sheep breeds in Nigeria. This study complements our recent molecular characterization of the mitochondrial *D-loop* region in Nigerian sheep [20]. The information obtained will be useful for designing appropriate breeding and selection schemes for indigenous sheep improvement and sustainable conservation.

## Results

### Morphological characterization

The basic descriptive statistics of the morphological traits of WAD, Yankasa, Uda and Balami sheep are presented in Table 1. The biometric traits of Uda and Balami sheep were significantly (p < 0.05) higher than those of WAD and Yankasa, although the latter had superior mean values than the former for all morphometric traits with the exception of EL. The effects of sex and system of management on the body parameters of the four sheep breeds are presented in Tables 2 and 3. Male animals had significantly higher body parameters than their female counterparts and higher values are observed in Balami and Uda compared with other breeds. The sexual dimorphism observed could be attributed to differences in the genetic architecture of the sheep populations. Animals reared intensively also had superior means than those semi-intensively managed for all morphological traits. While all the sheep breeds are reared semi intensively, only WAD and Yankasa are reared extensively. However, WAD is not reared intensively. The effect of age on morphological parameters was significant with slight increases with age (Table 4). Table 5 shows the spread of the four Nigerian sheep breeds across the country. WAD is only found in the southern part of the country. Variation was observed in the various morphological parameters studied according to breed in the different sampling location. The stepwise discriminant procedure showed that TL, RH, CG, EL and CD were the most discriminating variables to separate WAD, Yankasa, Uda and Balami sheep based on their significance and partial R^2^ values ≥0.01 (Tables 6 and 7, respectively). The canonical variate analysis (Table 7) clearly showed distinctive differences in the morphological traits of the four sheep breeds. Table 8 shows the percentage of individual sheep classified into genetic groups. The highest value is between WAD and WAD (93.33) and the lowest is between Uda and Uda (45.16). Error level is lowest in Yankasa (0.361). Results for kinship coefficient (Dkf) and proportion of shared alleles are presented in Table 9. The highest kinship coefficient and number of shared alleles is between WAD and Balami.1

**Table 1:** Descriptive statistics of the morphological traits of Nigerian sheep breeds

**Trait**	**WAD sheep**	**Yankasa sheep Uda sheep Balami sheep**		
	**LSM ± SEM**	**LSM ± SEM**	**LSM ± SEM**	**LSM ± SEM**
BW	20.12 ± 1.23^d^	30.87 ± 1.05^c^	37.86 ± 1.20^a^	39.00 ± 0.94^a^
WH	52.35 ± 1.07^d^	68.52 ± 0.6^c^	74.53 ± 0.87^a^	74.31 ± 0.59^a^
RH	52.12 ± 0.87^d^	67.44 ± 0.63^c^	73.78 ± 0.82^a^	73.64 ± 0.57^a^
BL	76.87 ± 1.53^d^	93.56 ± 0.91^c^	99.40 ± 0.99^a^	101.77 ± 1.21^a^
EL	13.03 ± 0.39^c^	13.19 ± 0.28^c^	15.63 ± 0.54^a^	14.54 ± 0.28^b^
FCL	13.45 ± 0.21^d^	14.85 ± 0.22^c^	16.53 ± 0.31^a^	15.60 ± 0.19^b^
TL	19.42 ± 0.63^d^	35.28 ± 0.54^c^	44.13 ± 0.73^a^	39.67 ± 0.50^a^
CG	65.04 ± 1.60^d^	75.83 ± 0.90^c^	81.30 ± 1.12^a^	82.87 ± 1.19^a^
CD	25.73 ± 0.78^d^	33.38 ± 0.45^c^	35.22 ± 0.58^a^	33.82 ± 0.32^a^
RW	11.84 ± 0.29^d^	13.77 ± 0.22^c^	15.35 ± 0.30^a^	15.32 ± 0.22^a^

a,b,c – means with different superscript are significantly (p < 0.05) different.

*BW* body weight, *WH* withers height, *RH* rump height, *BL* body length, *EL* ear length, *FCL fore cannon bone length*, *TL* tail length, *CG* chest girth, *CD* chest depth, *RW* rump width *WAD* West African Dwarf sheep, *LSM* least square means, *SEM* standard error of mean.

**Table 2:** Effects of sex on the morphological traits of Nigerian sheep breeds

	**Male**				**Female**			
	**Balami**	**Uda**	**WAD**	**Yankasa**	**Balami**	**Uda**	**WAD**	**Yankasa**
BW	40.23 ± 1.69	44.28 ± 1.86	27.01 ± 3.46	37.12 ± 1.83	37.49 ± 1.08	33.89 ± 1.53	17.37 ± 2.63	27.99 ± 1.25
BL	91.34 ± 3.92	102.06 ± 4.32	79.63 ± 8.02	89.67 ± 4.26	82.65 ± 2.51	96.01 ± 3.55	75.26 ± 6.10	91.34 ± 2.90
TL	45.00 ± 1.09	46.37 ± 1.20	21.27 ± 2.23	35.39 ± 1.18	44.01 ± 0.69	42.64 ± 0.98	18.34 ± 1.69	34.74 ± 0.80
WH	73.89 ± 1.36	76.66 ± 1.50	53.09 ± 2.78	67.02 ± 1.48	71.16 ± 0.87	70.98 ± 1.23	47.07 ± 2.12	67.01 ± 1.00
RW	74.72 ± 1.07	77.73 ± 1.17	54.95 ± 2.19	68.00 ± 1.16	73.34 ± 0.68	71.68 ± 0.97	50.47 ± 1.66	66.45 ± 0.79
CG	78.39 ± 1.44	83.79 ± 1.59	63.59 ± 2.95	74.64 ± 1.57	78.25 ± 0.92	77.85 ± 1.31	60.21 ± 2.24	73.05 ± 1.07
CD	27.95 ± 1.85	35.70 ± 2.03	25.68 ± 3.78	32.05 ± 2.01	29.58 ± 1.18	30.96 ± 1.67	23.07 ± 2.88	30.35 ± 1.37
RH	14.44 ± 0.35	15.38 ± 0.39	11.36 ± 0.74	12.84 ± 0.39	14.91 ± 0.23	14.45 ± 0.32	11.52 ± 0.55	13.86 ± 0.26
FCL	14.70 ± 0.55	17.73 ± 0.60	14.05 ± 1.12	16.05 ± 0.59	12.78 ± 0.35	14.40 ± 0.49	13.10 ± 0.85	14.59 ± 0.41
EL	16.91 ± 0.52	18.84 ± 0.58	14.63 ± 1.07	15.42 ± 0.57	13.65 ± 0.33	13.93 ± 0.47	12.10 ± 0.82	12.51 ± 0.39

**Table 3:** Effect of management systems on the morphological traits of Nigerian sheep breeds

	**Semi intensive**				**Extensive**				**Intensive**			
	**Balami**	**Uda**	**WAD**	**Yankasa**	**Balami**	**Uda**	**WAD**	**Yankasa**	**Balami**	**Uda**	**WAD**	**Yankasa**
BW	34.18 ± 1.14	35.76 ± 1.20	21.54.19.44	30.65 ± 1.03	-	-	19.44 ± 3.77	26.00 ± 11.32	44.81 ± 1.45	48.58 ± 2.74	-	47.00 ± 8.00
BL	69.86 ± 2.39	95.43 ± 2.68	76.81 ± 5.14	90.23 ± 2.15	-	-	77.00 ± 7.85	110.00 ± 23.56	109.54 ± 3.01	112.17 ± 5.71	-	116.00 ± 16.66
TL	41.94 ± 0.72	43.13 ± 0.80	20.11 ± 1.54	34.71 ± 0.64	-	-	17.77 ± 2.36	38.00 ± 7.09	48.05 ± 0.90	48.76 ± 0.90	-	47.50 ± 5.02
WH	71.10 ± 0.95	73.03 ± 1.07	49.00 ± 2.05	66.99 ± 0.85	-	-	49.94 ± 3.13	65.00 ± 9.39	73.31 ± 1.20	74.41 ± 2.27	-	69.5 ± 6.64
RW	73.08 ± 0.74	73.03 ± 0.84	52.33 ± 1.61	66.84 ± 0.67	-	-	51.61 ± 2.45	69.00 ± 7.37	74.80 ± 0.94	78.23 ± 1.78	-	72.00 ± 5.21
CG	76.70 ± 0.98	78.86 ± 1.10	60.79 ± 2.12	73.44 ± 0.88	-	-	63.00 ± 3.24	74.00 ± 9.73	80.82 ± 1.24	86.58 ± 2.36	-	80.00 ± 6.88
CD	25.54 ± 1.24	32.06 ± 1.40	24.02 ± 2.68	30.82 ± 1.12	-	-	24.05 ± 4.09	32.00 ± 12.28	34.78 ± 1.57	36.58 ± 2.97	-	34.50 ± 8.68
RH	14.23 ± 0.23	14.26 ± 0.26	11.26 ± 0.513	13.50 ± 0.21	-	-	11.94 ± 0.78	15.00 ± 2.35	15.13 ± 0.48	17.41 ± 0.57	-	15.00 ± 1.66
FCL	12.21 ± 0.38	15.65 ± 0.43	13.52 ± 0.82	15.06 ± 0.34	-	-	13.27 ± 1.25	15.00 ± 3.77	15.13 ± 0.48	16.17 ± 0.91	-	14.50 ± 2.67
EL	15.03 ± 0.40	16.09 ± 0.45	13.04 ± 0.86	13.47 ± 0.36	-	-	13.00 ± 1.31	12.00 ± 3.94	13.91 ± 0.50	15.14 ± 0.95	-	11.50 ± 2.78

**Table 4:** Effects of age on the morphological traits of Nigerian sheep breeds

	**< 1 year**				**1 -2 years**				**2 – 3 years**				**3 – 4 years**				**5 – 6 years**			
	**Balami**	**Uda**	**WAD**	**Yankasa**	**Balami**	**Uda**	**WAD**	**Yankasa**	**Balami**	**Uda**	**WAD**	**Yankasa**	**Balami**	**Uda**	**WAD**	**Yankasa**	**Balami**	**Uda**	**WAD**	**Yankasa**
BW	33.66 ± 2.27	42.33 ± 4.64	13.50 ± 8.04	29.14 ± 4.29	30.86 ± 2.27	33.92 ± 2.68	15.54 ± 3.42	27.46 ± 2.14	37.53 ± 1.52	36.27 ± 1.87	25.92 ± 3.03	32.62 ± 1.59	44.89 ± 1.75	40.51 ± 2.04	22.16 ± 6.56	31.79 ± 2.01	48.11 ± 3.79	58.75 ± 8.04	-	28.80 ± 5.08
BL	77.68 ± 4.94	88.45 ± 10.09	74.00 ± 17.47	76.10 ± 9.34	67.12 ± 4.94	89.25 ± 5.82	70.45 ± 7.45	83.06 ± 4.67	77.11 ± 3.30	99.23 ± 4.06	81.07 ± 6.60	92.58 ± 3.46	103.44 ± 4.43	103.23 ± 4.43	82.66 ± 14.27	96.13 ± 4.36	121.00 ± 8.23	123 ± 17.47	-	102.68 ± 11.05
TL	43.47 ± 1.41	38.45 ± 2.89	19.75 ± 5.00	28.71 ± 2.67	42.48 ± 1.41	41.85 ± 1.66	18.09 ± 2.13	35.93 ± 1.33	42.05 ± 0.94	43.85 ± 1.16	20.67 ± 1.89	35.50 ± 0.99	47.07 ± 1.09	45.72 ± 1.27	18.16 ± 4.08	34.47 ± 1.25	51.11 ± 2.36	63.00 ± 5.00	-	35.50 ± 3.16
WH	68.08 ± 1.80	66.13 ± 3.68	46.00 ± 6.38	58.14 ± 3.41	67.05 ± 1.80	70.62 ± 2.12	47.50 ± 2.72	66.21 ± 1.70	72.25 ± 1.20	73.69 ± 1.48	50.89 ± 2.41	67.39 ± 1.26	75.32 ± 1.39	75.16 ± 1.62	50.50 ± 5.20	68.33 ± 1.59	880.33 ± 3.00	82.00 ± 6.38	-	71.64 ± 4.03
RW	70.45 ± 1.43	71.25 ± 2.92	50.00 ± 5.05	61.28 ± 2.70	70.02 ± 1.43	72.31 ± 1.68	49.04 ± 2.15	65.11 ± 1.35	74.50 ± 0.95	73.84 ± 1.17	54.50 ± 1.91	67.75 ± 1.00	76.62 ± 1.10	75.41 ± 1.28	53.66 ± 4.13	68.24 ± 1.26	76.88 ± 2.38	84.50 ± 5.05	-	68.54 ± 3.19
CG	74.16 ± 1.86	77.63 ± 3.79	56.80 ± 6.58	67.21 ± 3.51	71.53 ± 1.86	75.45 ± 2.19	58.22 ± 2.80	72.34 ± 1.75	79.19 ± 1.24	79.51 ± 1.53	63.07 ± 2.48	73.07 ± 1.30	83.10 ± 1.67	83.10 ± 1.67	68.83 ± 5.37	76.12 ± 1.64	101.00 ± 6.58	101.00 ± 6.58	-	77.72 ± 4.16
CD	31.69 ± 2.51	35.06 ± 5.12	22.25 ± 8.88	34.77 ± 4.74	22.84 ± 2.51	30.25 ± 2.96	22.36 ± 3.78	28.29 ± 2.37	28.29 ± 1.67	33.36 ± 2.06	25.64 ± 3.35	31.44 ± 1.75	30.92 ± 1.93	32.85 ± 2.25	23.83 ± 7.25	30.96 ± 2.22	36.66 ± 4.18	41.50 ± 8.81	-	32.54 ± 5.61
RH	14.04 ± 0.45	12.42 ± 0.92	9.50 ± 1.59	11.31 ± 0.85	13.15 ± 0.45	13.53 ± 0.53	11.00 ± 0.68	12.66 ± 0.42	14.62 ± 0.30	14.70 ± 0.37	11.78 ± 0.60	13.92 ± 0.31	16.36 ± 0.34	15.90 ± 0.40	13.00 ± 1.30	14.16 ± 0.39	15.33 ± 0.75	19.50 ± 1.59	-	13.64 ± 1.01
FCL	13.01 ± 0.76	14.76 ± 1.56	13.25 ± 2.71	15.18 ± 1.45	11.16 ± 0.76	14.49 ± 0.90	12.86 ± 1.15	14.60 ± 0.72	13.30 ± 0.51	16.38 ± 0.63	13.85 ± 1.02	15.35 ± 0.53	14.56 ± 0.59	15.99 ± 0.69	13.83 ± 2.21	14.67 ± 0.67	14.66 ± 1.28	14.50 ± 2.71	-	16.88 ± 1.71
EL	13.28 ± 0.79	15.61 ± 1.61	12.50 ± 2.80	12.14 ± 1.49	15.04 ± 0.79	14.55 ± 0.93	12.31 ± 1.19	13.53 ± 0.74	15.22 ± 0.52	16.33 ± 0.65	13.85 ± 1.05	14.01 ± 0.55	14.38 ± 0.61	16.23 ± 0.71	12.16 ± 2.28	12.61 ± 0.70	14.38 ± 1.32	16.50 ± 2.80	-	14.04 ± 1.77

**Table 5:** Effects of sampling location on the morphological traits of Nigerian sheep breeds

	**South**				**Middle belt**				**North East**				**North West**				**North Central**			
	**Balami**	**Uda**	**WAD**	**Yankasa**	**Balami**	**Uda**	**WAD**	**Yankasa**	**Balami**	**Uda**	**WAD**	**Yankasa**	**Balami**	**Uda**	**WAD**	**Yankasa**	**Balami**	**Uda**	**WAD**	**Yankasa**
BW	45.66 ± 6.37	43.00 ± 3.67	20.91 ± 2.01	44.26 ± 2.53	47.22 ± 3.67	46.99 ± 3.32	-	28.47 ± 1.74	32.79 ± 1.61	32.10 ± 2.08	-	23.00 ± 7.80	42.32 ± 1.32	40.15 ± 1.89	-	27.08 ± 2.67	34.18 ± 2.01	34.38 ± 3.18	-	29.15 ± 1.64
BL	102.66 ± 11.77	98.11 ± 6.79	76.86 ± 3.72	92.000 ± 4.67	110.09 ± 6.79	108.89 ± 6.14	-	98.94 ± 3.22	59.74 ± 2.95	102.47 ± 3.85	-	70.55 ± 14.42	108.43 ± 2.45	106.11 ± 3.49	-	99.76 ± 4.94	58.09 ± 5.88	58.09 ± 5.88	-	80.60 ± 3.04
TL	44.00 ± 4.03	41.74 ± 2.32	19.41 ± 1.27	36.36 ± 1.60	43.78 ± 2.32	44.50 ± 2.10	-	31.47 ± 1.10	44.06 ± 1.01	43.71 ± 1.32	-	44.50 ± 4.93	47.51 ± 0.84	45.85 ± 1.19	-	37.47 ± 1.69	37.47 ± 1.27	41.84 ± 2.01	-	36.05 ± 1.04
WH	74.66 ± 5.19	77.88 ± 3.00	49.28 ± 1.64	65.23 ± 2.06	79.29 ± 3.0	82.01 ± 2.71	-	68.95 ± 1.42	67.62 ± 1.31	70.92 ± 1.70	-	55.50 ± 6.36	73.56 ± 1.08	72.67 ± 1.54	-	69.52 ± 2.18	72.59 ± 1.64	69.05 ± 2.59	-	65.60 ± 1.34
RW	77.00 ± 4.17	78.33 ± 2.40	52.11 ± 1.31	68.39 ± 1.65	77.57 ± 2.40	79.60 ± 2.17	-	67.69 ± 1.14	73.25 ± 1.05	71.65 ± 1.36	-	62.75 ± 5.11	74.88 ± 0.87	74.20 ± 1.23	-	69.58 ± 1.75	70.42 ± 1.31	71.53 ± 2.08	-	64.85 ± 1.07
CG	76.50 ± 5.58	78.92 ± 3.22	61.45 ± 1.76	72.26 ± 2.21	82.05 ± 3.22	86.60 ± 2.91	-	75.60 ± 1.52	75.57 ± 1.41	78.87 ± 1.82	-	71.00 ± 6.83	80.32 ± 1.16	81.79 ± 1.65	-	78.05 ± 2.34	76.94 ± 1.76	74.31 ± 2.79	-	70.70 ± 1.44
CD	33.66 ± 6.71	33.33 ± 3.87	24.03 ± 2.12	30.15 ± 2.66	36.56 ± 3.87	41.26 ± 3.50	-	32.72 ± 1.83	25.31 ± 1.69	32.65 ± 2.19	-	48.00 ± 8.22	34.37 ± 1.40	35.58 ± 1.99	-	33.94 ± 2.82	20.24 ± 2.12	17.72 ± 3.35	-	27.67 ± 1.73
RH	15.16 ± 1.35	14.61 ± 0.78	11.46 ± 0.42	12.86 ± 0.53	15.51 ± 0.78	15.65 ± 0.70	-	14.08 ± 0.37	13.84 ± 0.34	14.42 ± 0.44	-	12.05 ± 1.65	15.39 ± 0.28	16.02 ± 0.40	-	13.35 ± 0.56	14.55 ± 0.42	11.80 ± 0.67	-	13.47 ± 0.35
FCL	20.00 ± 1.75	20.88 ± 1.01	13.45 ± 0.55	17.21 ± 0.69	18.95 ± 1.01	20.37 ± 0.91	-	16.48 ± 0.48	11.41 ± 0.44	16.62 ± 0.57	-	15.60 ± 2.15	14.78 ± 0.36	14.55 ± 0.52	-	13.94 ± 0.73	10.68 ± 0.55	8.98 ± 0.87	-	13.28 ± 0.45
EL	19.83 ± 2.06	20.33 ± 1.19	13.03 ± 0.65	16.26 ± 0.82	18.83 ± 1.19	21.46 ± 1.07	-	13.12 ± 0.56	14.77 ± 0.52	15.75 ± 0.67	-	12.00 ± 2.52	13.62 ± 0.43	13.89 ± 0.61	-	11.52 ± 0.86	14.48 ± 0.65	13.64 ± 1.03	-	13.30 ± 0.53

**Table 6:** Stepwise selection of traits

**Traits entered**	**Partial R**^**2**^	**F value**	**Pr > F**	**Wilk’s lambda**	**Pr < lambda**	**ASCC**	**Pr > ASCC**
Tail length	0.4913	128.12	<0.0001	0.508710	<0.0001	0.164	<0.0001
Rump height	0.1056	15.63	<0.0001	0.454978	<0.0001	0.183	<0.0001
Chest girth	0.1055	15.49	<0.0001	0.356692	<0.0001	0.246	<0.0001
Ear length	0.0650	9.18	<0.0001	0.425392	<0.0001	0.201	<0.0001
Chest depth	0.0626	8.79	<0.0001	0.398760	<0.0001	0.212	<0.0001

*ASCC* Average squared canonical correlation.

**Table 7:** Total canonical structure of the discriminant analysis of the four sheep breeds

**Traits**	**CAN1**	**CAN2**	**CAN3**
Rump height	0.839	0.098	0.038
Chest girth	0.493	−0.112	−0.159
Chest depth	0.485	0.261	−0.012
Tail length	0.922	−0.107	0.098
Ear length	0.239	−0.242	0.791

**Table 8:** Percentage of individual sheep classified into genetic group

**Breed**	**WAD**	**Yankasa**	**Uda**	**Balami**
WAD	93.33	6.67	0.00	0.00
Yankasa	1.64	63.93	15.57	18.85
Uda	0.00	17.20	45.16	37.63
Balami	0.00	17.83	21.02	61.15
Error level	0.067	0.361	0.548	0.389
Priors	0.250	0.250	0.250	0.250

**Table 9:** Kinship coefficient (Dkf) below the diagonal and proportion of shared alleles above the diagonal between the Nigerian sheep breeds

	**Balami**	**Uda**	**WAD**	**Yankasa**
Balami	0.000	0.757	0.800	0.366
Uda	0.941	0.000	0.481	0.643
WAD	0.946	0.789	0.000	0.693
Yankasa	0.861	0.906	0.915	0.000

### Molecular genetic diversity between populations

Polymorphism Information Content (PIC) and F statistics (F_*IS*_, F_*IT*_, F_*ST*_) according to Weir and Cockerham (1984), G_*ST*_ and Shannon index values for all 15 microsatellite markers analyzed in Nigerian sheep breeds are shown in Table 10. The l5 microsatellite loci demonstrated high polymorphism in this population with PIC values ranging from 0.751 to 0.927 (Table 10) lending strong support to the use of this panel of markers for assessing genetic diversity in Nigerian sheep.

**Table 10:** **Polymorphism Information Content (PIC), F statistics (*F***_***IS***_ ***, F***_***IT***_ ***, F***_***ST***_**) according to Weir and Cockerham (1984) *G***_***ST ***_**and shannon index values for 15 microsatellite markers analyzed in Nigerian sheep breeds**

**Locus**	**PIC**	***F***_***IS***_	***F***_***IT***_	***F***_***ST***_	***G***_***ST***_	**Shannon index**
DYMS1	0.927	0.442	0.454	0.022	0.125	2.964
OarCP34	0.751	0.373	0.436	0.099	0.106	2.203
OarFCB193	0.845	0.220	0.329	0.140	0.163	2.321
BM8125	0.808	0.316	0.416	0.146	0.130	2.082
OarJMP29	0.904	0.287	0.352	0.092	0.084	2.854
OarJMP58	0.899	0.318	0.361	0.063	0.079	2.325
OarFCB128	0.782	0.534	0.593	0.126	0.187	2.212
OarFCB304	0.901	0.319	0.339	0.029	0.033	2.882
SRCRSP1	0.787	0.251	0.301	0.066	0.046	2.263
OarAE129	0.792	0.325	0.365	0.059	0.130	1.653
OarVH72	0.853	0.171	0.278	0.129	0.142	2.375
SRCRSP5	0.896	0.406	0.472	0.111	0.175	2.914
MCM 140	0.872	0.428	0.459	0.055	0.069	2.332
OarHH 47	0.825	0.387	0.436	0.079	0.211	2.172
SRCRSP9	0.857	0.245	0.320	0.099	0.122	2.268
Mean	0.846	0.335	0.394	0.088	0.120	2.388

Differences in the values of global F_*ST*_, F_*IT*_ and F_*IS*_ over all loci (Table 10) considered in this study shows the suitability of some microsatellite markers over the others in the study of genetic diversity in Nigerian sheep breeds. The mean value of F_*ST*_ is 0.088 while that of F_*IT*_ and F_*IS*_ are 0.394 and 0.335 respectively. The highest value of F_*ST*_ (0.146) was observed for BM8125 while the lowest value of 0.022 was seen in DYMS1. Inbreeding values within and across breeds can also be attributed to selection. F_*ST*_ and G_*ST*_ are indices of population subdivision. Global F_*ST*_, G_*ST*_ and G_*ST’*_ over all loci were all significantly different (p < 0.001). The population differences examined by global analysis of F_*ST*_ (coefficient of multilocus genetic differentiation fixation index) for each of 15 microsatellite loci across the four sheep breeds revealed that most of the total genetic variation corresponds to differences among individuals within breeds (91.2%) and 8.8% result from differences among breeds. Values of G_*ST*_ ranged from 0.033 for OarFCB304 to 0.211 for OarHH47 with a mean of 0.120 (Table 10). The results of G_*ST*_ in this study reveal that gene variation among the breeds is still low. This differentiation formed the basis for describing how genetic variation is partitioned within Nigerian sheep breeds. Among the loci considered in this study, OarFCB304 had the highest Shannon information index value of 2.51 and the least index value of 1.391 was observed for OarHH47.

Number of effective alleles, allelic richness, expected heterozygosity and observed heterozygosity are presented in Table 11. Effective number of alleles ranged from 17.330 in Yankasa to 7.200 in WAD. This trend was also observed for allelic richness with Yankasa having a value of 10.51 and WAD, 6.59. Yankasa had the highest expected heterozygosity (0.849) while WAD had the least (0.684). Mean values for observed heterozygosity across the breeds ranged from 0.405 in Uda to 0.563 in Yankasa (Table 11).

**Table 11:** Effective number of alleles (Na), Allelic Richness, Expected Heterozygosity (He) and Observed Heterozygosity (Ho) in Nigerian sheep breeds

**Breed**	**Effective No. of alleles (Na)**	**Allelic richness**	**Expected Heterozygosity**	**Observed heterozygosity**
Balami	13.730	9.240	0.823	0.558
Uda	9.000	8.200	0.754	0.405
WAD	7.200	6.590	0.684	0.448
Yankasa	17.330	10.510	0.849	0.563
Mean	11.815	8.635	0.778	0.494

**Figure 1:**
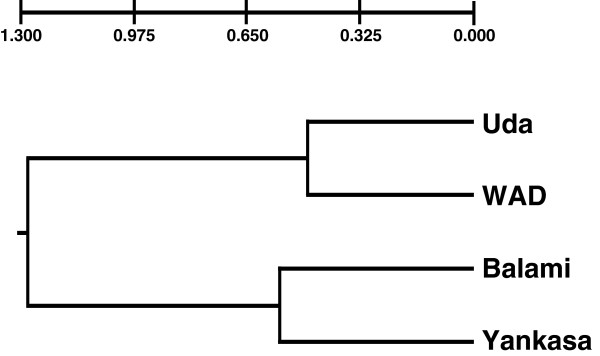
Dendogram showing diversity and similarity among Nigerian breeds of sheep.

The analysis of molecular variance (AMOVA) results revealed that the greatest variation (60.716%) is within individuals, 30.545% among individuals within populations and 8.739 among populations which are consistent with the F_*ST*_ results (Table 12). Figure 1 presents the genetic distances (DA) between Nigerian sheep breeds.

**Table 12:** AMOVA design and results (average over 15 loci)

**Source of variation**	**Sum of squares**	**Variance components**	**Percentage variation**
Among populations	172.808	0.585	8.739
Among individuals within populations	1432.042	2.04	30.545
Within individuals	759.500	4.063	60.716
Total	2364.350	6.692	

**Figure 2:**
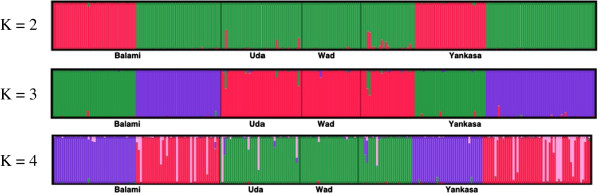
**Population structure assessed by Structure. **Bar plot, generated by DISTRUCT, depicts classifications with the highest probability under the model that assumes independent allele frequencies and inbreeding coefficients among assumed clusters. Each individual is represented by a vertical bar, often partitioned into colored segments with the length of each segment representing the proportion of the individual’s genome from K = 2, 3, 4 ancestral populations. Breeds are separated by black lines.

Population structure assessed by STRUCTURE software and barplots generated by DISTRUCT are presented in Figure 2. Classifications with the highest probability under the model that assumes independent allele frequencies and inbreeding coefficients among assumed clusters revealed the presence of ancestral populations (K) which is consistent with the morphological and diversity analyses reported earlier in this study. At K = 2, two clusters were constituted from breeds descended from Balami and Yankasa, both of which are from Northern Nigeria. At K = 3 and K = 4, one more cluster emerged and further analyses did not reveal any additional strong high level substructure, so separating the entire dataset into 3 major clusters was chosen as the final configuration. There are however, several cases of admixtures in the genome of some of the individuals that constitute the cluster. Yankasa and Balami breeds had more cases of admixtures followed by Uda while the WAD breed had the least cases of admixtures.

## Discussion

### Morphological diversity

Phenotypic characterization has been shown to be an accessible and easy-to-use tool in conservation and breeding programs [6]. This could be explained in part by the high heritability of measures of size across ontogeny [21]. Marked differences were observed in the morphological traits of the sheep breeds in this study. The present values of BW and linear body measurements of Yankasa, Uda and Balami sheep are higher than the range of values reported for Ganjam sheep in India by Arora et al. [10]. Most researchers consider an organism’s phenotype as a multivariate set of variables and the covariation of traits an important analytical consideration [22]. Discriminant analysis of morphometric traits is a mathematical approach that has been widely used in determining the relationships between different breeds of livestock [6,23-25]. The most discriminating variables obtained in this study are similar to previous reports by Dossa et al. [16] and Vargas et al. [26]. The present results indicate that there is significant morphological differentiation among Nigerian sheep populations. This morphological diversity pattern could be as a result of inherent genetic potential of each breed, alongside geographical isolation, ecological variation and community isolation [5,27]. This is noticeable in the remarkable morphological differences between WAD (more adaptable to the wet, dense forest and derived savannah zones of southern Nigeria) and Yankasa, Uda and Balami sheep (more suited to the dry climatic conditions of Northern Nigeria).

The larger values reported for the conformation traits of males are in consonance with earlier reports on sheep [28], goats [26] and cattle [29]. However, Bacchi et al. [30] found no sexual dimorphism in the morphometric characters considered in *Lama guanicoe guanicoe* in Argentina. The higher values recorded for intensively managed animals might be as a result of better nutrition and management practices as they tend to gain more attention than those reared semi-intensively. Riva et al. [14] reported that husbandry system was a source of variation in the body measurements of Bergamasca sheep; theirs was a case between transhumance and sedentary systems, where the former was taller, longer and showed a wider hock while the latter was wider at the chest and rump.

The three canonical functions (linear combinations of the continuous variables that summarize variation between the four sheep breeds) obtained could be used as criterion for establishing phenotypic standards for Nigerian sheep. The closeness between Uda and Balami sheep compared to their WAD and Yankasa counterparts might be as a result of near biometric convergence, which may function as a guide to genetic and evolutionary relationships between the two breeds. The longer distance between WAD and other breeds, especially Balami and Uda revealed that phenotypic differences are maintained in part by the reduction of gene flow among populations separated by large distances as well as physical-ecological barriers. The WAD sheep predominate in the trypano-endemic humid zones of Southern Nigeria. The intermediate morphology of Yankasa sheep might be maintained by natural selection on body size of individuals inhabiting intermediate or a range of different environments (this breed of sheep, which occupies a central geographical position, is more widely distributed in the country than the other three sheep breeds). Selective advantage might therefore favor Yankasa sheep from the biogeographical context, although more heterotic gains might be attained from crosses involving WAD and Uda or Balami. This is because populations are dynamic units which adapt physiologically and genetically to their environments and sensitive to, and within limits, responsive to any change in their environmental conditions. An appreciable percentage of animals were classified into their distinct breeds. However, some level of intermingling was observed between Uda and Balami, which could partly be attributed to indiscriminate crossbreeding due to geographical proximity.

### Molecular genetic diversity between populations

The high number of alleles observed in Yankasa must have contributed to its adaptability to more agro-ecological zones in Nigeria compared to other sheep breeds that may confer selective advantages [19]. Genetic variation is necessary to allow organisms to adapt to ever-changing environments with some of this variation stemming from introduction of new alleles by the random and natural process of mutation, since the frequency of occurrence of an allele changes regularly as a result of mutation, genetic drift, and selection [31]. The number of alleles identified in this study is slightly higher than those reported by Adebambo et al. [19] with observed number of alleles in Yankasa, Balami, Uda and WAD being 11.5, 3.9, 5.85 and 5.05, respectively, based on relatively small sample sizes. Higher numbers of alleles may also be reflective of larger sample sizes used in this study. However, the values fall within the range reported for other sheep breeds. Arora *et al.*[10] reported values for observed number of alleles in the range of 7 and 25 with a mean value of 13.96. The small average number of alleles per locus in Uda and WAD may be the result of a small number of founder animals since the degree of genetic variability among these animals will be small even in the presence of crossbreeding and mutation rates [32].

Different indices used in this study demonstrate the existence of genetic diversity among Nigerian sheep breeds. Variations in allelic richness were observed across all loci. Mean values among the various loci ranged from 3.66 in OarHH47 to 14.73 in OarJMP29, with mean values of 8.63. Dalvit et al. [33] found similar values of allelic richness with an average of 8.8, when they analyzed 10 European sheep breeds. Measures of genetic diversity based on allelic richness are considered important in conservation genetics because marker-assisted methods for maximizing the number of alleles conserved have been shown to be effective [34]. Allelic richness may be a useful indicator of a decrease in population size or of past bottlenecks [35]. It is also relevant in a long-term perspective, as selection limits are determined by the initial allelic composition rather than by heterozygosity [36]. As noted by El Mousadik & Petit [37] the often reported numbers of alleles per locus for the whole population or averaged over subpopulations are not comparable because of the much larger sample size of the whole population.

Differences among populations are commonly quantified by the use of one of several statistics, including Wright’s inbreeding coefficient (F_*ST*_) and Nei’s coefficient of gene variation (G_*ST*_) [38]. The rather high level of genetic variability in Yankasa is due to the fact that this is the most numerous sheep breed in Nigeria [19]. These values (0.658 to 0.902) are within the range observed in other sheep breeds in other parts of the world. Arora et al. [10] observed 0.594 to 0.922 for sheep breeds from Southern Peninsular and Eastern regions of India. Values for Italian sheep breeds are 0.761 to 0.805 [39] and European sheep breeds ranged from 0.538 to 0.807 [40]. The observed heterozygosity values are generally lower than the expected heterozygosity in all the breeds and loci considered. The highest value of observed heterozygosity in Yankasa could also be attributed to its large number in Nigeria [19]. The results of G_*ST*_ analysis in this study reveal that the proportion of gene variation among the breeds is still low.

The Shannon index of Nigerian sheep breed in this study revealed low species richness and evenness since all the indices were below 3.5, the mark set for high species evenness and richness [41]. This might not be unconnected with the level of heterozygote deficiency observed among this population, possibly due to the management system [42]. Data collected within the EU-ECONOGENE project on sheep and goat diversity in marginal areas indicate the presence of significant inbreeding in most of the breeds [7,43]. This is likely due to poor breeding management of frequently small herds, which leads to partial isolation and fragmentation both at the local and breed levels [44].

Differences in the values of global *F*_*ST*_, *F*_*IT*_ and *F*_*IS*_ over all loci considered in this study supports the suitability of some microsatellite markers over the others in the study of genetic diversity in Nigerian sheep breeds. Selection based on estimated breeding values rather than phenotypic merits can lead to the extensive use of a small number of elite individuals in purebred livestock populations, and could potentially increase inbreeding [45]. It has therefore been suggested that breeds with wide range of genetic diversity are needed in the future for generating transgressive variation for quantitative trait loci mapping and developing new genotypes for particular management systems and market demands [46]. On the average, within-breed heterozygote deficit (F_*IS*_) was observed to be 33.5% whereas the total population (F_*IT*_) exhibited 39.5% deficit with significant values (p < 0.05). Some investigations have reported relatively low variability in local non-selected breeds, as is the case of certain Nigerian [19] and Chinese sheep [47].

The relative higher genetic identity is probably due to the continuous crossing between populations at least in the recent past [48]. The AMOVA results revealed that the greatest variation (60.716%) is within the individual, 30.545% among individuals within populations and 8.739 among populations which are consistent with *F*_*ST*_ results. Finally, the trend in genetic relationship between these Nigerian sheep breeds is the same for delta mu square, kinship coefficient and proportion of shared alleles. Indeed, our recent molecular characterization of Nigerian sheep using mtDNA sequences of the *D-loop* found 96 haplotypes, but only 5 haplotypes are common to all the breeds [20], showing relatively divergent haplotypes within breeds and geographical locations. This suggests that gene flow has occurred on a regional scale at some time in the recent past and that the breeds have not been subdivided by long term biogeographic barriers.

### Genetic structure of the populations

The closest genetic distance between Yankasa and Balami at 0.184 and the farthest distance between Balami and WAD (0.665) may be reflective of their geographical locations in Nigeria since Yankasa and Balami are more of northern than southern breeds. This is further confirmed by the greater genetic distance between WAD and Balami observed in this study. These results are supported by Adebambo et al. [19] among these breeds. The pattern of differentiation revealed by the matrix of Nei’s genetic distances and the tree topology reflected the evolutionary history, geographical distributions and the gene flow among breeds. Genetic structure of a breed at any time is the result of a balance between genetic drift (founder effect and selection) and gene flow [32]. WAD and Balami shared the highest number of alleles while the least is between Yankasa and Balami. However, the pattern of genetic distances deviated from the morphological distances obtained for the four sheep breeds. This may be related to varying sensitivity of the two distance estimates, although both methods were able to separate the southern WAD goats from their northern counterparts. A similar finding was reported in Ankole cattle [49] where the results of morphological analyses were not in concordance with the molecular genetic relationship results. The authors attributed this to the fact that microsatellites loci are selectively neutral whereas morphological traits are under selection.

Results from the STRUCTURE analysis revealed that varying the number of presumed ancestral populations (K) produces clusters that are consistent with the observed morphological categorization. The first level of clustering (K = 2) reflects the presence of two clusters in the four breeds examined and further evaluation revealed a third strong cluster. This result suggests that the four breeds originated from three ancestral populations which diverged as a result of several years of adaptation and domestication. Further evaluation of the clusters revealed the presence of sub clusters and admixtures which are indicative of substantial gene flow between these breeds. WAD is the only breed with a minimal case of admixture. This may be due to geographical delineation of the breed and the breeding practices of the owners of these animals predominantly in the southern part of the country. Yankasa possess alleles which are shared by the other three breeds and this is consistent with the results discussed above.

The mitochondrial data showed a different relationship in the neighbor-joining tree for the four Nigerian sheep breeds [20]. In that tree, the first divergence was for Yankasa breed, followed by WAD and later by Uda and Balami. This can be explained by differences in the breeding patterns, by the use of dams and rams in different management schemes. The higher level of concordance of morphological and mitochondrial DNA data could mean a more ancestral relationship among the breeds revealed by mitochondrial DNA that is maternally inherited. Differences found in the microsatellite data may indicate recent crossbreeding due to geographical closeness among the sympatric breeds, especially involving males from one breed crossing with females of the other breeds.

## Conclusion

Morphological and genetic diversity of Nigerian sheep breeds is eroding gradually. These results suggest that within-breed genetic variation observed in Nigeria sheep is more than between-breed and this variation could be a valuable tool for genetic improvement and conservation. The higher genetic variability in Yankasa may mean the presence of unique alleles reflecting the presence of certain functional genes which may possibly be related to better adaptability of Yankasa in more agro-ecological zones of Nigeria. The higher level of heterozygosity in this study provides the basis for further improvement through selection of primarily Yankasa as well as the other breeds.

## Methods

### Study area and population

**Figure 3:**
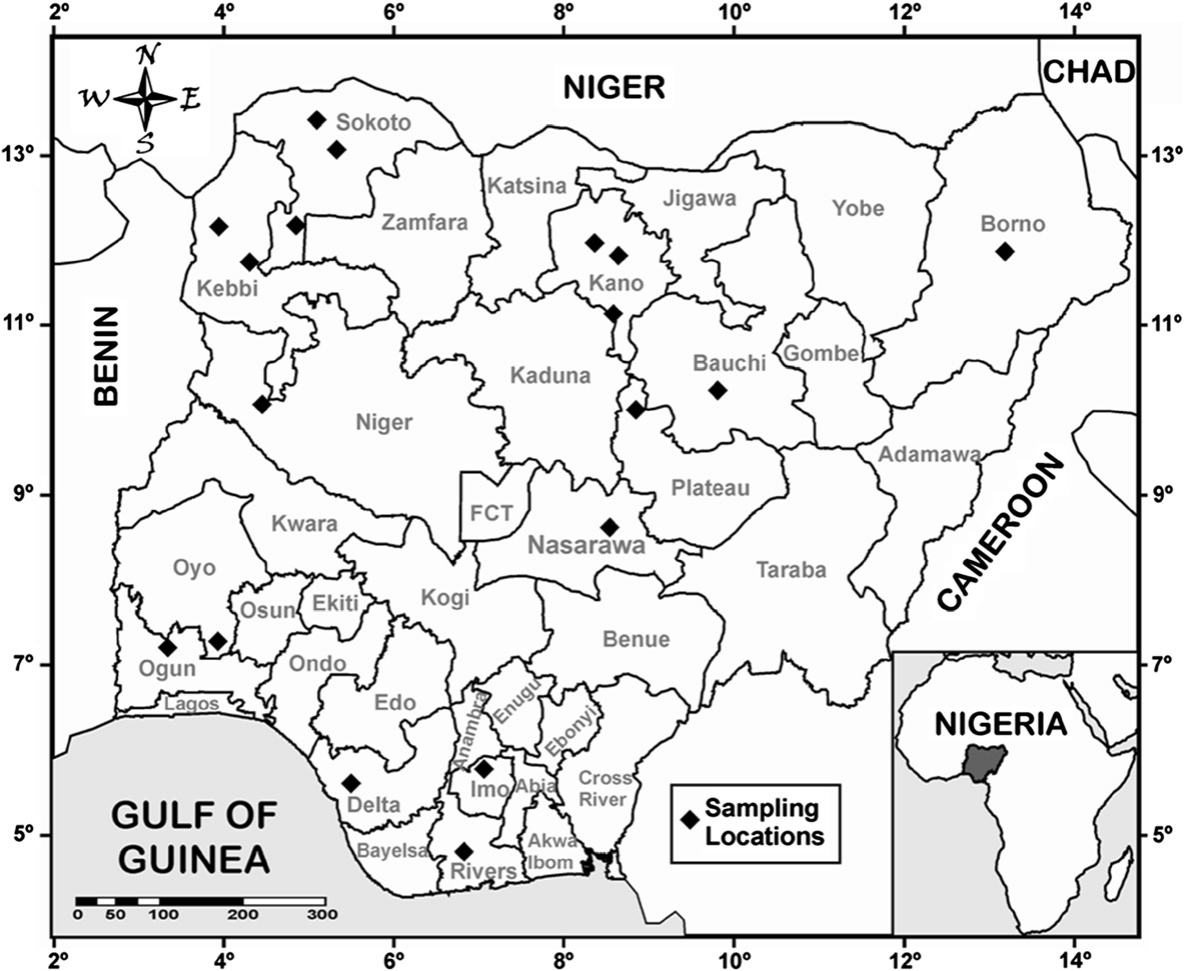
Map of Nigeria showing sampled locations.

Samples were collected across Nigeria covering all agro-ecological zones from the arid North to the dense rainforest bordering the coast in the South. Nigeria is located in West Africa on the Gulf of Guinea (latitude 10º00’ N, longitude 8º00’ E) with a total area of 923,768 km^2^ (twice the size of California). Nigeria is bounded by Niger, Benin and Cameroon Republics on the North, West and East, respectively (Figure 3). The protocol for the experiment was approved by the Institutional Animal Use and Care Committee of the University of Agriculture, Abeokuta (UNAAB), Nigeria. A random sample of 402 sheep (132 males and 270 females) of the four Nigerian sheep breeds [Balami – 133, Uda – 94, West African Dwarf (WAD) – 52, Yankasa – 123] were selected from cities and villages across the country. There is no relationship between sires and dams of the animals sampled since they came from different parts of the country. The animals were reared in semi-intensive (323 animals) and intensive (79 animals) systems of management. They were at least 15.5 months of age (2-tooth to 8-tooth age) and where records were missing; age was estimated using dentition as described by Wilson and Durkin [50]. Body weight (BW) (kg) and nine body linear measurements (cm), namely height at withers (HW), rump height (RH), body length (BL), ear length (EL), fore cannon bone length (FCL), tail length (TL), chest girth (CG), chest depth (CD) and rump width (RW) respectively were taken on each animal, following standard procedure and anatomical reference points earlier reported [24,28]. In addition, 5–7 ml of whole blood were collected from 384 sheep of the four breeds [Balami – 106, Uda – 94, West African Dwarf (WAD) – 52, Yankasa – 132] into heparinized tubes from the jugular vein of each animal and stored on ice before they were transferred to the laboratory for analysis.

### DNA extraction, polymerase chain reaction (PCR) and fragment analysis

DNA was extracted from 50 μl of whole blood using the ZymoBead™ Genomic DNA Kit (Zymo Research Corp. Irvine, CA, USA) according to the manufacturer’s recommendations and DNA yield and quality were assessed using a Nanodrop ND-100 Spectrophotometer (Nanodrop Technologies, Inc., DE, USA). The DNA was amplified by PCR in a MyCycler™ Thermal Cycler (Biorad, Hercules, CA, USA) using 15 microsatellite markers selected from the FAO recommended list [51] described in Table 13. The 20 μl amplification reactions contained 2 μl containing 30-50 ng template DNA, 2.0 μl of each primer, and 16 μl nuclease free water in a AccuPower® TLA PCR Premix containing NTPs, MgCl_2_ and *Taq* DNA polymerase (Bioneer Corp., Irvine, CA, USA) using annealing temperatures shown in Table 13. PCR protocol was as follows: denaturing at 94°C for 5 minutes, 35 cycles of amplification at 94°C for 30 seconds, annealing at annealing temperature of marker for 30 seconds, extension at 72°C for 1 minute, final extension at 72°C for 5 minutes and held at 4°C until analysis. PCR products were separated by electrophoresis in 1.5% agarose gel stained with 0.5 μg/ml ethidium bromide. Electrophoresis was carried out at room temperature for 1 hour at 100 volts using a Bio-Rad Power Pac™ electrophoresis apparatus (Biorad, Hercules, CA, USA). The resulting amplified bands were visualized with UV light and photographed using the Alphalmager™ 2200 gel documentation and analysis system (Cell Biosciences, CA, USA), and were scored using GENEMate Quanti-Marker 100 bp DNA ladder (BioExpress, Kaysville, UT, USA).

**Table 13:** Microsatellite primer sequences that were used and their base lengths

	**Name**	**Sequence**	**Base length**	**Annealing temperature (°C)**	**Allele range (bp)**	**Dye**
1	OarFCB193	TTCATCTCAGACTGGGATTCAGAAAGGC	28	54	96-136	6FAM
		GCTTGGAAATAACCCTCCTGCATCCC	26			
2	OarJMP29	GTA TAC ACG TGG ACA CCG CTT TGT AC	26	56	96-150	NED
		GAA GTG GCA AGA TTC AGA GGG GAA G	25			
3	OarJMP58	GAAGTCATTGAGGGGTCGCTAACC	24	58	145-169	6FAM
		CTTCATGTTCACAGGACTTTCTCTG	25			
4	OarFCB304	CCCTAGGAGCTTTCAATAAAGAATCGG	27	56	150-188	6FAM
		CGCTGCTGTCAACTGGGTCAGGG	23			
5	OarAE129	AATCCAGTGTGTGAAAGACTAATCCAG	27	54	133-159	6FAM
		GTAGATCAAGATATAGAATATTTTTCAACACC	32			
6	BM8125	CTCTATCTGTGGAAAAGGTGGG	22	50	110-130	VIC
		GGGGGTTAGACTTCAACATACG	22			
7	OarFCB128	ATTAAAGCATCTTCTCTTTATTTCCTCGC	29	55	96-130	VIC
		CAGCTGAGCAACTAAGACATACATGCG	27			
8	OarCP34	GCTGAACAATGTGATATGTTCAGG	24	50	112-130	VIC
		GGGACAATACTGTCTTAGATGCTGC	25			
9	OarVH72	GGCCTCTCAAGGGGCAAGAGCAGG	24	57	121-145	VIC
		CTCTAGAGGATCTGGAATGCAAAGCTC	27			
10	OarHH47	TTTATTGACAAACTCTCTTCCTAACTCCACC	31	58	130-152	VIC
		GTAGTTATTTAAAAAAATATCATACCTCTTAAGG	34			
11	DYMS1	AACAACATCAAACAGTAAGAG	21	59	159-211	NED
		CATAGTAACAGATCTTCCTACA	22			
12	SRCRSP1	TGC AAG AAG TTT TTC CAG AGC	21	54	116-148	NED
		ACC CTG GTT TCA CAA AAG G	19			
13	SRCRSP5	GGA CTC TAC CAA CTG AGC TAC AAG	24	56	126-158	NED
		GTT TCT TTG AAA TGA AGC TAA AGC AAT GC	29			
14	SRCRSP9	AGA GGA TCT GGA AAT GGA ATC	21	55	99-135	6FAM
		GCA CTC TTT TCA GCC CTA ATG	21			
15	MCM140	GTT CGT ACT TCT GGG TAC TGG TCT C	25	60	167-193	NED
		GTC CAT GGA TTT GCA GAG TCA G	22			

DNA fragment analysis of microsatellite markers was carried out using the Applied BioSystems 3730xl DNA Analyzer (Applied Biosystems, Carlsbad, CA, USA) at the Cornell University Genomics Core Facility. GeneMapper Software version 3.0 [52] (which combines the functions of GeneScan and Genotyper software in one convenient package) was used to generate microsatellite genotypes.

### Data analysis

Means, standard deviations, standard errors and coefficients of variation were computed for all the traits measured using the GLM procedure of SAS statistical package [53]. Sources of variation in the linear model were breed, sex and system of management. Multivariate analysis [discriminant analysis] was employed to investigate morphological structure, and quantify differences among the sheep populations. Stepwise discriminant analysis was performed to gain information about the most important traits in separating the four sheep breeds using the STEPDISC procedure. These most important variables were then subjected to canonical discriminant analysis using the CANDISC procedure to derive canonical functions and estimate Mahalanobis distances necessary for the differentiation of the sheep populations. The ability of these canonical functions to allocate individual sheep to its original breed was calculated as percentage correct assignment of each breed using the DISCRIM procedure (Nearest Neighbour Discriminant Analysis with Mahalanobis Distances) of the SAS statistical package. Microsatellite Analyzer (MSA) version 4.05 [54] and GENEPOP [55] were used to generate genetic diversity parameters.

A model-based Bayesian clustering analysis was used to infer population structure and the level of admixture in the sheep breeds implemented in STRUCTURE v2.3 [56]. The STRUCTURE algorithm assumes K populations, each of which is in Hardy-Weinberg and linkage equilibria and characterized by a set of allele frequencies at each locus. Analysis was performed with a burn in length of 20,000 followed by 100,000 Markov chain Monte Carlo iterations for each of K = 1 to 6, with ten replicate runs for each K using independent allele frequencies and an admixture model. Results across ten runs at each K were compared based on similarity coefficients (SC) as previously described [57]. The breeds were assigned to wide clusters based on major ancestry and submitted to a second round of STRUCTURE analysis performed within each wide cluster.

## Abbreviations

BL: Body length; BW: Body weight; CD: Chest depth; CG: Chest girth; EL: Ear length; FCL: Fore cannon bone length; HW: Height at withers; RH: Rump height; RW: Rump width; TL: Tail length.

## Competing interests

The authors declare that they have no competing interests.

## Authors’ contributions

IGI and BOA conceived the study; BOA, IGI, OAA, MOO, CONI designed the study; IGI and MO obtained funding for the study; BOA, MAA, AY, MW, TMS, OOA, BMI, SAA, GOO and JOE collected phenotypic data and contributed to morphological data analysis; BOA, AY, and OOA carried out morphological data analysis; BOA, MAA and MD carried out the molecular lab analysis; BOA, MAA, SOP, MD, KK and IGI carried out the molecular data analysis and interpretation, BOA, MAA, IGI, MO, AY and SOP wrote the manuscript. All authors read and approved the manuscript.
